# Untangling the R2* contrast in multiple sclerosis: A combined MRI-histology study at 7.0 Tesla

**DOI:** 10.1371/journal.pone.0193839

**Published:** 2018-03-21

**Authors:** Francesca Bagnato, Simon Hametner, Emma Boyd, Verena Endmayr, Yaping Shi, Vasiliki Ikonomidou, Guanhua Chen, Siddharama Pawate, Hans Lassmann, Seth Smith, E. Brian Welch

**Affiliations:** 1 Neuroimaging Unit/ Neuroimmunology Division, Department of Neurology, Vanderbilt University Medical Center; Nashville, TN, United States of America; 2 Department of Neuroimmunology, Center for Brain Research, Medical University, Vienna, Austria; 3 Department of Bioengineering, George Mason University, Fairfax, VA; 4 Department of Biostatistics, Vanderbilt University School of Medicine, Nashville, TN, United States of America; 5 Department of Biostatistics and Medical Informatics, University of Wisconsin School of Medicine and Public Health, Madison, WI, United States of America; 6 Neuroimmunology Division, Department of Neurology, Nashville, TN, United States of America; 7 Vanderbilt University Institute of Imaging Science, Department of Radiology and Radiological Sciences, Vanderbilt University Medical Center, Nashville, TN, United States of America; Henry Ford Health System, UNITED STATES

## Abstract

T2*-weighted multi-echo gradient-echo magnetic resonance imaging and its reciprocal R2* are used in brain imaging due to their sensitivity to iron content. In patients with multiple sclerosis who display pathological alterations in iron and myelin contents, the use of R2* may offer a unique way to untangle mechanisms of disease. Coronal slices from 8 brains of deceased multiple sclerosis patients were imaged using a whole-body 7.0 Tesla MRI scanner. The scanning protocol included three-dimensional (3D) T2*-w multi-echo gradient-echo and 2D T2-w turbo spin echo (TSE) sequences. Histopathological analyses of myelin and iron content were done using Luxol fast blue and proteolipid myelin staining and 3,3′-diaminobenzidine tetrahydrochloride enhanced Turnbull blue staining. Quantification of R2*, myelin and iron intensity were obtained. Variations in R2* were found to be affected differently by myelin and iron content in different regions of multiple sclerosis brains. The data shall inform clinical investigators in addressing the role of T2*/R2* variations as a biomarker of tissue integrity in brains of MS patients, in vivo.

## Introduction

T2*-weighted (T2*-w) multi-echo gradient-echo (ME-GRE) magnetic resonance imaging (MRI) and its reciprocal R2* relaxation rate [R2* = 1/T2*] are sensitive to the presence of iron in tissue of normal and diseased brains [1–10]. Iron produces T2* signal decay through its paramagnetic effect on susceptibility and microscopic field gradients [1,10]. This effect increases with field strength, making ME-GRE at 7.0 Tesla (7T) exquisitely sensitive for imaging iron in tissue [11–13]. When imaging human brains, however, one cannot infer that all areas of T2* signal decay only correspond to the presence of iron. T2* signal decay may occur virtually in every brain region but more profoundly so in regions with different magnetic susceptibility such as heterogeneous tissues or at boundaries, i.e., tissue-tissue, tissue-bone and tissue-air [1]. Orientation of white matter (WM) fiber bundles relative to the magnetic field [14–17], macroscopic geometry [18,19], variations in myelin content [20–22], and calcium [23,24] are also possible sources of T2* signal decay. Myelin can also enhance T2* relaxation due to its diamagnetism and water fraction, especially in WM regions with high myelin and low iron content such as the optical radiations [25]. Diamagnetic and anisotropically structured myelin results in microscopic orientation-dependent field variations [25] and may increase R2* by as much as 60% in vivo [26,27]. Diamagnetic frequency contrast in WM relative to gray matter (GM) is observed after iron extraction, suggesting that iron-free myelinated WM is more diamagnetic than iron-free GM [4]. In animals with experimental demyelination a significant increase of T2* in WM and a marginal increase of T2* in GM is observed compared to healthy animals confirming that myelin is a fundamental source of T2* contrast [27].

Multiple sclerosis (MS) is an inflammatory, neurodegenerative disease of the central nervous system whereby myelin loss is a cardinal pathological feature [28]. In MS, myelin loss is a potent contributor to T2* signal decay. However, several pathological MS features may cause changes in iron content and/or myelin geometry, ultimately contributing to T2* signal decay. These pathological features include: (1) remyelination, that results in altered myelin architecture [29]; (2) iron-rich macrophages scavenging myelin debris and toxic products liberated during demyelination [30]; and (3) neurodegeneration associated with expression of inducible nitric oxide synthase in microglia [31] and profound accumulation of iron at a subset of slowly expanding lesion edges [7, 32]. Given the potentially complex interplay between myelin and iron concentration and R2* values in MS brain tissue, in the present study, we performed side-by side comparisons between staining for iron, myelin and R2*-w ME-GRE MRI. We then obtained measurements of iron and myelin concentrations as well as R2* and assessed the strength of the association between histology and MRI measures as function of different brain regions, normal-appearing and diseased. Based on theoretical considerations of relaxivity and prior literature [33], we hypothesized an overall association between iron/myelin and R2* values across samples and regions, with the strength of this association varying as a function of quality and quantity of the underlying MS pathology within individual samples.

## Materials and methods

### Study design

This combined imaging-pathological post mortem study is a collaborative project between Vanderbilt University (Nashville, TN, U.S.), the Medical University of Vienna (Austria) and George Mason University (Fairfax, VA, U.S.). The study was performed under local ethics approval as required by each institution. The institutional review board of the Medical University of Vienna approved the study (EK number 535/2004). The U.K. MS Society Tissue Bank [34] supplied tissue samples according to a peer-reviewed research protocol and inter-institutional material transfer agreement. All potential donors and their next-of-kin completed and signed a consent form during life. At the time of signing the consent, donors were fully capable of understanding its content. The consent was given for use of tissue for research in MS and other neurological conditions. Although later on during histopathological analysis, Alzheimer's pathology was observed, this represented an incidental finding during routine neuropathology and was not mirrored by clinical manifestations. Therefore, the studied cases were not Alzheimer's disease cases per se and did not come from a vulnerable population.

Eight coronal brain slices deriving from seven brains of patients with secondary progressive MS and one with primary progressive MS designated MS-1 to MS-8 hereafter were used. We report demographic, clinical and pathological features of each donor in Table 1.

**Table 1:** Demographic and clinical features of donors.

Case	Years of age / Sex	Cause of death	Disease duration (years)	Years since progressive MS / wheelchair	Co-existent pathology
**MS-1**	72 / Female	Sepsis—meningitis—MS	33	18 / 7	None
**MS-2**	90 / Female	Carcinomatosis—lung cancer—MS	33	29 / Unknown	AD type *tau* pathology
**MS-3**	83 / Female	Pneumonia—CHF—AKF	47	13 / 12	None
**MS-4**	74 / Female	Breast cancer stage 4—MS	48	28 / 20	Amyloid angiopathy
**MS-5**	88 / Female	CVA—MS	30	24 / 13	AD’s like changes
MS-6*	88 / Female	Aspiration pneumonia	36	34 / Unknown	AD’s like changes
**MS-7**	55 / Male	MS	31	26 / 15	None
**MS-8**	66 / Male	Bronchopneumonia	22	19 / 17	None

AD: Alzheimer disease; AKF: acute kidney failure; CHF: congestive heart failure; CVA: cerebrovascular accident.

* PPMS

### Tissue collection

The Multiple Sclerosis Society Tissue Bank (funded by the Multiple Sclerosis Society of Great Britain and Northern Ireland) provided tissue samples used for this study. For each subject, a single 1-cm thick coronal slice was donated and a choice was made to select the one passing though thalamic and deep gray matter (dGM) regions.

A single coronal cut through the mammillary bodies separated the brain into anterior and posterior halves. Thereafter, 10-mm thick coronal slices through the entire brain were obtained. Brain slices were fixed in 4% paraformaldehyde at 4°C for several years after variable lengths of post mortem intervals (4–83 hours). The coronal slice through the basal ganglia and lateral ventricles of each brain were used for our study. For imaging, slices were immersed in 4% neutral-buffered paraformaldehyde inside a cylindrical, specially fabricated tissue container [35].

### Imaging

#### MR protocol

Scans were acquired using a whole-body 7T Achieva MRI scanner (Philips Healthcare, Cleveland, OH) equipped with a volume transmit and 32-channel receiver head coil (NOVA Medical, Wilmington, MA). The scanning protocol included three-dimensional (3D) T2*-w ME-GRE and two-dimensional (2D) T2-w turbo spin echo (TSE) sequences [36]. An average image of repeated acquisitions was created in order to increase the signal-to-noise ratio. For each acquisition, 40 contiguous slices of .7 mm^3^ isotropic resolution were acquired. Pulse sequence details are reported in Table 2.

**Table 2:** Pulse sequence parameters.

	2D T2-w TSE	T2*-w ME-GRE
Field of view (cm × cm)	18 × 24	16 × 16
Repetition time (ms)	23,000	4,000
Turbo Spin Echo factor	4	Not applicable
Echo time(s)	45	2.9 / 20.9 / 38.9 / 56.9 / 74.9
Flip Angle	90	90
Repetitions	2	9
Number of slices	40	40
Voxel size (mm × mm × mm)	0.70 × 0.72 × 0.70	0.70 × 0.70 × 0.70
Sense factor	2	None
Imaging plane	Coronal	Coronal
Scan time (hours:minutes:seconds)	0:25:18	2:16:52

#### ME-GRE reconstruction

T2* maps were reconstructed by the scanner based on the signal decay of the multi-echo magnitude images. The T2* fitting algorithm implemented on the scanner is an advanced method [37] that accounts for static magnetic field gradients in the through-plane direction while finding a single optimal mono-exponential T2* decay value. Specifically, the fitting method accounts for sinc function modulations induced by large-scale magnetic field variations. T2* maps were converted to R2* maps using the mathematical relationship, R2* = 1/T2*.

T2* maps were registered to the T2-w TSE using a linear registration with 7 degrees of freedom, as implemented in the FLIRT routine of the FSL library.

#### MR images’ analysis and ROI placement and quantification

Images were visualized using the Medical Image Processing, Analysis, and Visualization application (MIPAV version 7.2.0; http://mipav.cit.nih.gov). Image analysis was performed blinded to the histopathology results. One investigator (EB) manually traced regions of interest (ROIs) on R2* maps using graphic tools available in MIPAV. To avoid partial volume effects, care was taken so that: (1) the size of the ROI was always ≥10 voxels [38] (4.9 mm^2^) and (2) each ROI was positioned away from boundary regions, e.g., at the interface of GM with WM or normal with diseased tissue. For each slice, ROIs were placed in areas of the brain displaying different features based on MRI (R2*) appearance and were distributed with an approximately equal number in the right and left hemispheres whenever possible. ROI were placed on MRI slices for which whole hemisphere histology was available. Each ROI mask was reviewed by a senior investigator (FB) for accuracy. ROIs were positioned on the R2* map, but anatomical localization was verified against the T2-w TSE sequence (which was acquired with the same geometric off-center and angulation) and myelin staining. The following measurements were derived: number of voxels and mean value of R2* (expressed in reciprocal seconds, s^-1^).

### Histochemistry and immunohistochemistry

Histochemistry and immunohistochemistry used in this study have been previously described [32]. Briefly, double-hemispheric tissue sections were cut with a tetrander microtome at a thickness of 10 μm and mounted on large glass slides. For basic classification of pathology and demyelination, sections were stained with Hematoxylin and Eosin (H&E) and Luxol fast blue (LFB) myelin staining (Division Chroma). PLP delivers a good intracortical myelin contrast which is better than that observed with LFB [39]. Therefore, PLP intensities were used to evaluate and analyze ROIs within the cortex. Iron was detected with 3,3′-diaminobenzidine-tetrahydrochloride (DAB) enhanced Turnbull blue staining [30]. Immunohistochemical staining for myelin (proteolipid protein, PLP) was performed with a primary anti-human PLP monoclonal mouse antibody (# MCA839G, AbD Serotec, Oxford, UK). Sections were steamed for 60 minutes in EDTA buffer pH 8.5. The primary antibody was diluted 1:1000 in 10% fetal calf serum in Dako wash buffer (FCS/DAKO) and applied at 4°C overnight. The next day, an appropriate biotinylated secondary anti-mouse antibody was applied. After incubation of the slides with avidin-conjugated horseradish peroxidase, the stainings were developed with DAB. Sections were coverslipped with Eukitt.

#### Histologic ROIs placement and definition

By histology, ROIs could be characterized for precise anatomical location and accurate pathological definition. In the WM, we considered the following: normal appearing WM (NAWM, ROIs placement in internal capsula, corpus callosum, and throughout the deep WM of each hemisphere), diffuse WM injury (DWMI, equivalent to diffusely abnormal WM), centers of WM lesions (WM-Ls, which were either completely inactive or slowly expanding), and shadow plaques (which were those WM-Ls expressing some degree of remyelination). DWMI were defined as areas of widespread myelin and axonal injury [28]. These areas displayed a global reduction in the intensity of myelin staining due to decreased fiber density (axons and myelin) but without degradation of fiber orientation. Shadow plaques were defined as sharply demarcated lesions with reduced myelin density and a moderate reduction of axonal density, as previously described [40,41]. In the GM, we delineated ROIs in deep and cortical structures. In the normal-appearing deep GM ROis were delineated in the substantia nigra, putamen, nucleus ruber, nucleus caudatus, globus pallidus internus and globus pallidus externus. These areas were cumulatively referred as dGM. We also delineated ROIs in the thalamus and the normal-appearing cortical grey matter, the latter defined as cortex throughout this paper.

#### Matching between MRI and histology

**Fig 1:**
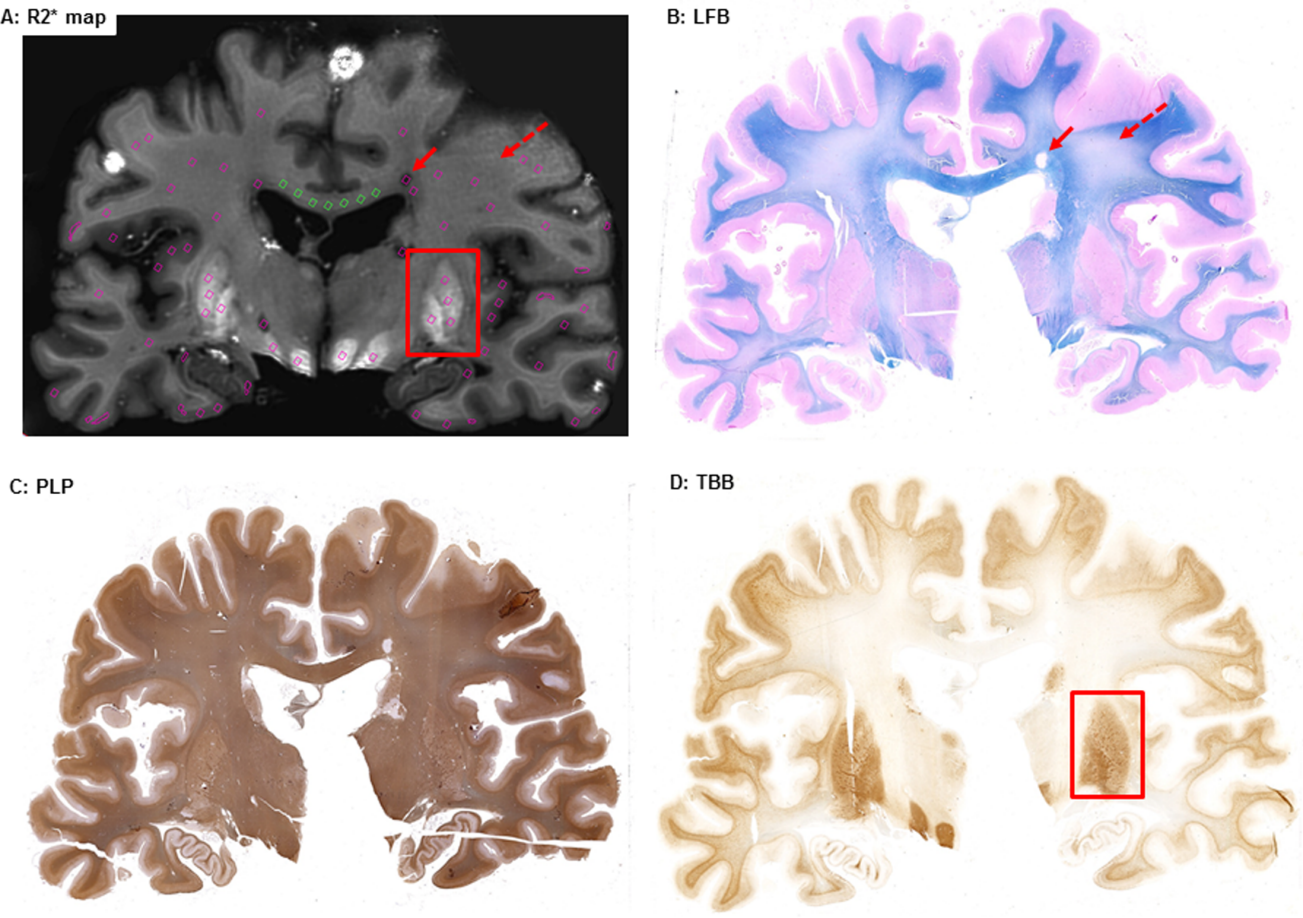
Side-by-side R2* (A), LFB (B), PLP (C) and TBB (iron, D) staining. One can appreciate examples of ROIs showing WM-L (solid red arrow on PLP and R2* maps), DWMI (dashed red arrow on PLP and R2* maps), and areas of increased iron accumulation in the dGM (red rectangle on TBB and R2* maps).

Fig 1 exemplifies side-by-side an MRI map containing the ROIs, and histological stains.

Two senior investigators (FB and SH) compared side-by-side histology and MRI ROI placement to confirm that each manually drawn ROI corresponded to the desired area on the histology staining.

#### Histological quantification

High-resolution images (2560 x 1920 pixels; 0.76 x 0.57 mm) of iron and myelin (LFB, PLP) stains of the lesion centers and adjacent NAWM were obtained with a Nikon DS-Fi1® digital camera mounted on a Reichert Polyvar 2® microscope with the 10x objective using the Nikon NIS Elements® software version 3.10. For this purpose, images were acquired under standardized conditions by controlling the lamp brightness and camera white balance before each imaging session when no slide was under the objective. Iron and myelin integrated density values were determined using ImageJ. To separate blue LFB from pink periodic acid Schiff (PAS) staining in RGB images of LFB-PAS sections, a color deconvolution plug-in (freeware kindly provided by A.C. Ruifrok, NIH) was run using the vector H-PAS. Resulting blue channels containing the dissected LFB myelin signal were converted into 8-bit grey scale images. PLP stains were color de-convoluted using the vector H-DAB, and resulting red channels contained the dissected DAB signal of the PLP. As the sections stained for iron were not counterstained, color deconvolution was dispensable and iron RGB images were directly converted into grey scale images. For all types of stains, the summation of all 8-bit values yielded the overall staining intensity over the image, termed “LFB intensity”, “iron intensity” and “PLP intensity”, reflecting variations in the diffuse reactivity of the stains.

### Statistical analysis

Continuous data were summarized using the median (25^th^, 75^th^ percentile) and categorical data were summarized using percentage. To assess differences in R2*, myelin (LFB intensity) and iron (iron intensity) contents among different ROI types, measurements were averaged for each ROI and each subject and were analyzed using the mixed effects model to take consideration the within subject correlation. Model based, pairwise comparisons were performed among ROIs. The false discovery rate (FDR) procedure was employed for correction of multiplicity.

To quantify associations between R2* values and histology measurements, two types of analyses were performed. With the primary analysis, we hypothesized that the association between R2* and iron/myelin intensity could vary depending upon the region and we calculated individual within subject (sample) Pearson correlation coefficients and their standard errors using a fixed effects meta-analysis method as previously described [42]. This approach was used to address the large degree of data correlation within each subject. The overall correlation coefficient was then provided by the weighted average of the individual correlation coefficients, where the weights were the relative precisions, i.e., the inverse of the standard error estimates of the individual coefficients. In the presented figures, data are shown for those subjects with ≥2 observations per ROI (in order to calculate valid correlation coefficients). The secondary analysis included linear mixed effects models and examined the marginal (overall) associations between R2* and LFB / iron intensity after adjusting for anatomical region due to the potential confounding effect. Subject was treated as a random effect to take into consideration the within-subject correlations. All analyses were implemented using R 3.3.0 (R Foundation for Statistical Computing, Vienna, Austria). A p-value ≤.05 was used for statistical significance.

## Results

A total of 429 ROIs were delineated and analyzed. ROI sizes ranged between 11–52 voxels (mean ± SD / median: 20.8 ± 4.2 / 20). Histological ROIs were location-matched to MRI ROIs but constant in size (0.43 mm^2^). Table 3 reports the total number and percentage of ROIs for each anatomical, healthy or pathologically altered area.

**Table 3:** ROIs distribution.

ROI location	Number of ROIs	Percent (%) of ROIs
**NAWM, n = 129**		
Corpus callosum	51	11.9
Internal capsula	12	2.8
Others	66	15.4
**GM (n = 185)**		
Cortex	114	26.6
**dGM, n = 71**		
Globus pallidus externus	18	4.2
Globus pallidus internus	6	1.4
Nucleus caudatus	4	0.9
Nucleus ruber	6	1.4
Putamen	28	6.5
Substantia nigra	9	2.0
**Mixed Normal GM/WM ROIs**		
Thalamus	15	3.5
**Pathologically affected areas, n = 100**		
WM-Ls	13	3.0
Shadow plaques	8	1.8
DWMI	79	18.4

dGM = deep gray matter; DWMI = diffuse white matter injury; GM = gray matter; NAWM = normal appearing white matter; WM-Ls = white matter lesions.

### Differences in R2* values, myelin and iron content among different anatomical regions

**Fig 2:**
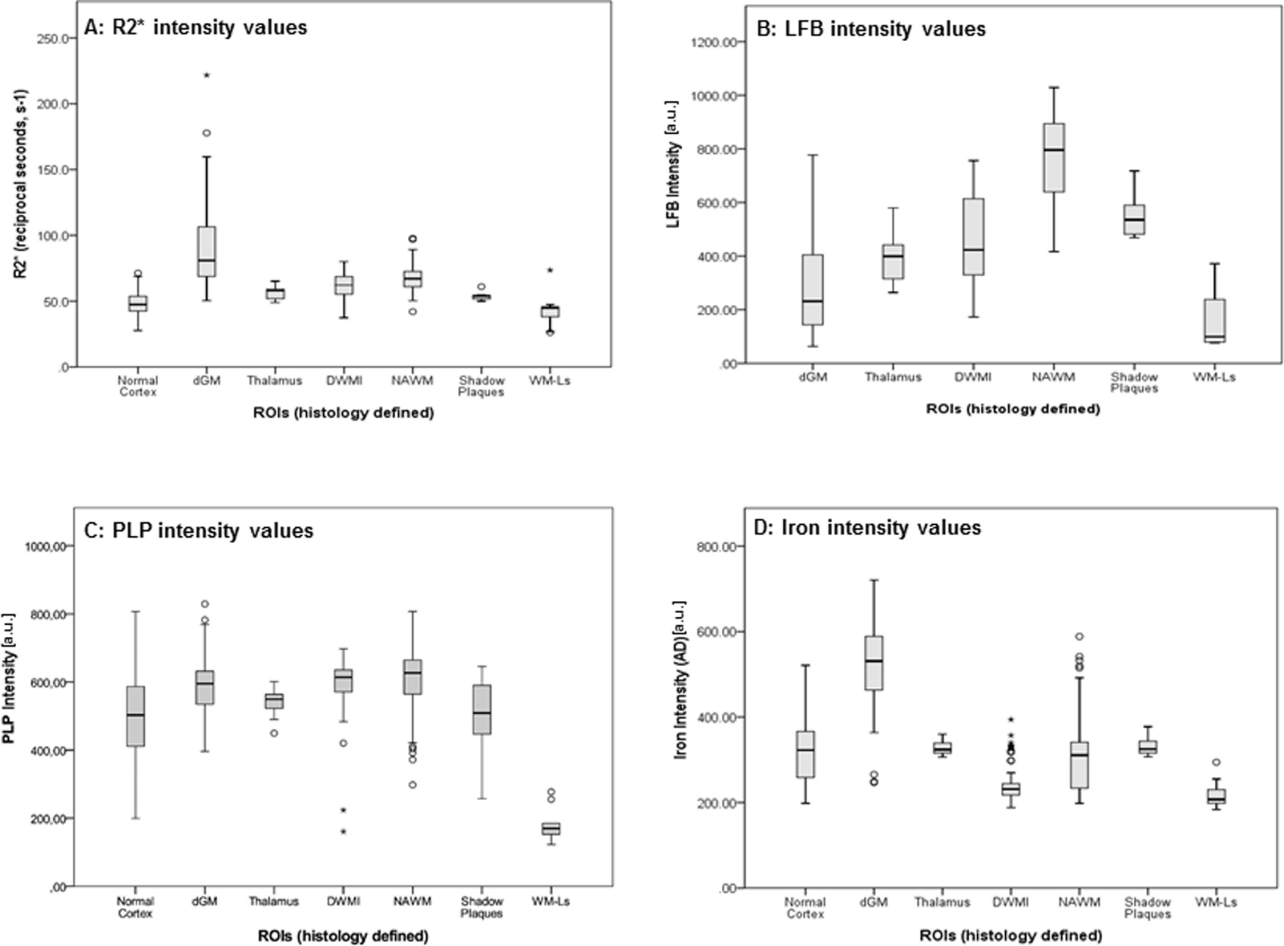
Differences in R2* values (A), LFB (myelin, B), PLP (myelin, C) and iron intensities (D) among different ROIs. Boxes represent the 25^th^-75^th^ percentile (interquartile range, IQR) and the black line inside the boxes represents the median value. The whiskers delineate all values within 1.5x IQR away from the respective lower or upper quartile, while circles (10^th^-90^th^) and stars (5^th^-95^th^) represent outliers. In the fig: dGM = deep gray matter, DWMI = diffuse white matter injury, NAWM = normal white matter, WM-Ls = white matter lesions (central core).

Fig 2A–2D depicts boxplots of R2*, myelin (LFB and PLP) and iron intensities of the examined ROIs. Statistically significant differences in R2* values, myelin and iron intensities were seen between several ROIs as detailed below.

#### R2* values (Fig 2A)

R2* values in the dGM ROIs were the highest and higher than those measured in the cortex, thalamus, NAWM, DWMI (p < .001 for all comparisons), shadow plaques (p = .002), and WM-Ls (p < .001). R2* values in NAWM were the second highest and higher than those measured in the cortex (p < .001) and WM-Ls (p < .001). WM-Ls also showed lower R2* values than those in the DWMI (p = .019).

#### Myelin content: LFB (Fig 2B) and PLP intensities (Fig 2C)

For myelin content, differences were examined only among LFB intensities measured in all regions but the cortex (see methods for explanation). LFB of the NAWM was the highest and higher compared to shadow plaques (p = .017), DWMI, WM-Ls, thalamus and dGM (p < .001 for all comparisons). WM-Ls also had lower LFB intensity measures than shadow plaques (p = .001) and DWMI (p < .001). The latter, however, had higher LFB intensity values than dGM ROIs (p = .005). No formal analyses were done with data derived from the PLP staining in WM regions. Data inspection, however, showed that PLP intensities were highest in the NAWM and similarly high in areas of DWMI. Thus in DWMI myelin reduction was less apparent with PLP than LFB staining. Shadow plaques showed a partial loss of PLP reactivity, while in WM-Ls PLP was almost completely lost. PLP intensities in the dGM and thalamus were close to the values observed in the NAWM, while values in the normal cortex were lower.

#### Iron content: iron intensity (Fig 2D)

Iron intensity in the dGM ROIs was the highest and higher than that measured in the cortex, thalamus, NAWM, DWMI, shadow plaques and WM-Ls (p < .001 for all comparisons). Iron intensities in NAWM were higher only than those detected in WM-Ls (p = .016). WM-Ls iron intensities had the lowest median of all regions and were lower than those in normal cortex (p < .001).

### Associations between R2* values and myelin/iron content in different anatomical regions within-samples: primary analyses

**Fig 3:**
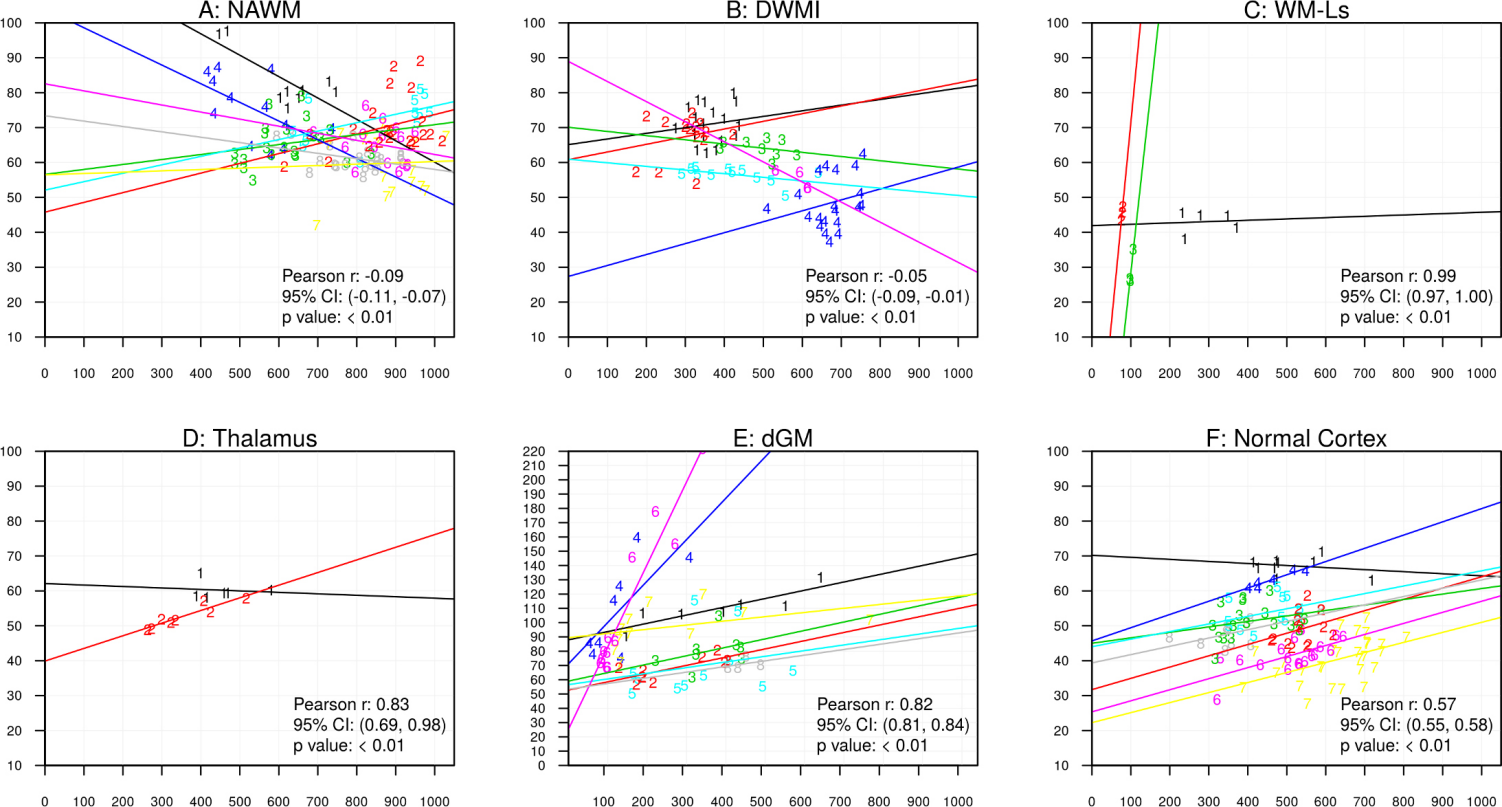
Scatter plots of R2* values and myelin intensity in ROIs placed in NAWM (A, LFB), DWMI (B, LFB), WM-Ls (C, LFB), thalamus (D, LFB), dGM (E, LFB) and cortex (F, PLP). Data points are labeled with subject ID number, so that the same number indicates multiple measurements from the subject. Y-axes indicate R2* values and x-axes indicate myelin concentrations. In the fig: au = arbitrary units; dGM = deep gray matter, DWMI = diffuse white matter injury, NAWM = normal appearing white matter, WM-Ls = white matter lesions. Each different colored line represents data from a different sample.

**Fig 4:**
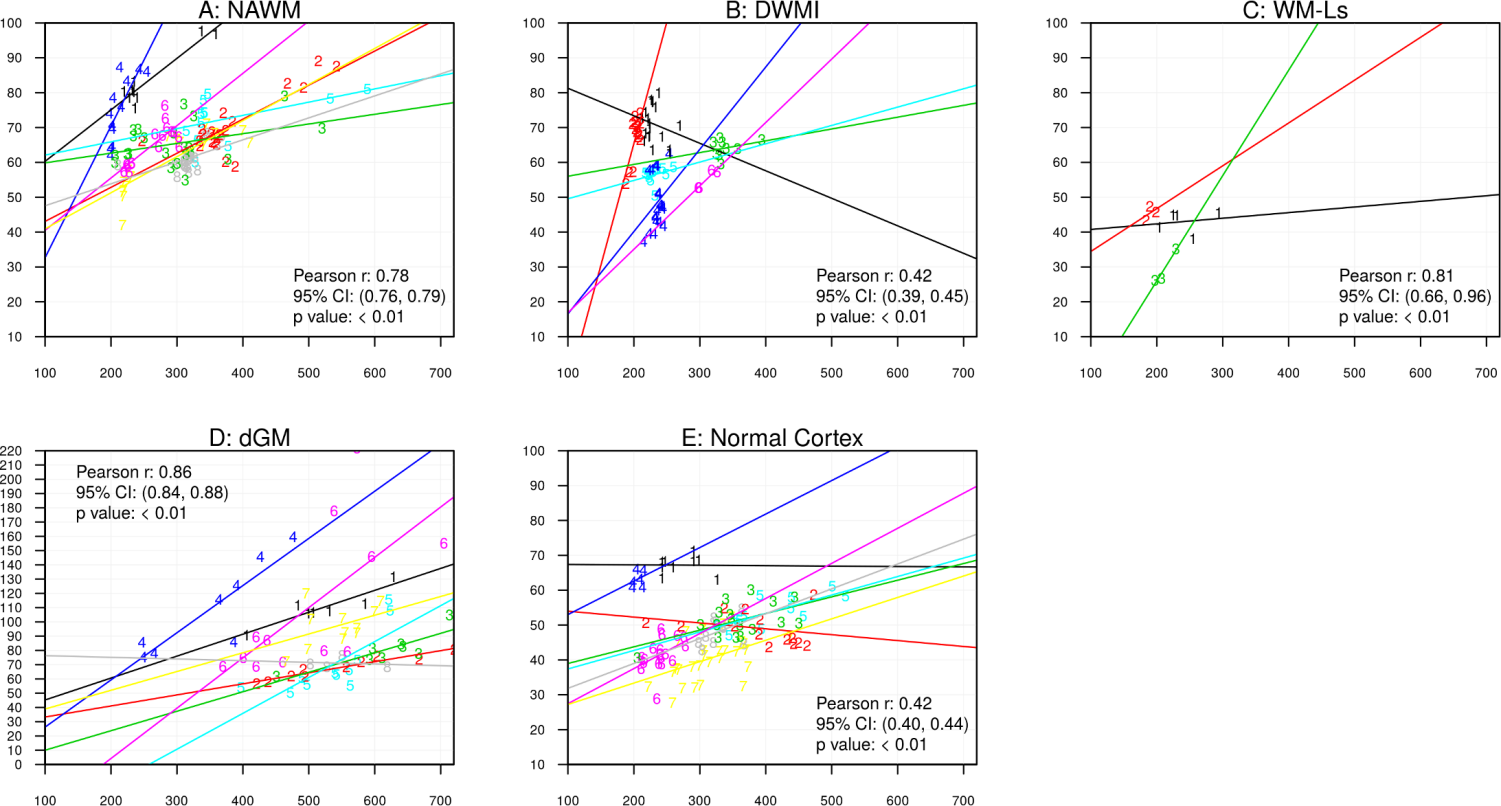
Scatter plots of R2* values and iron intensity in ROIs placed in NAWM (A), DWMI (B), WM-Ls (C), dGM (D) and cortex (E). Data points are labeled with subject ID number, so that same number indicates multiple measurements from the subject. Y-axes indicate R2* values and x-axes indicate iron concentrations. In the fig: au = arbitrary units; dGM = deep gray matter, DWMI = diffuse white matter injury, NAWM = normal white matter, WM-Ls = white matter lesions. Each different colored line represents data from a different sample.

Figs 3 and 4 show associations between MRI and histology data. In these figures, we display only significant associations for those subjects that had ≥2 observations. Overall Pearson correlation coefficients r (calculated by consolidating individual within-subject correlation Pearson correlation coefficients), 95% confidence interval (CI) and p-values are presented.

#### R2* values and myelin (LFB intensity, Fig 3A–3E, PLP intensity Fig 3F)

LFB intensities were inversely associated with R2* values in ROIs of NAWM (A, p < .01, overall Pearson coefficient = -0.09, 95% CI: −0.11, −0.07) and DWMI (B, p < .01, overall Pearson coefficient = -0.05, 95% CI: −0.09, −0.01). Although significant, these associations were weak since resulting from overall Pearson coefficient averaged over positive and negative individual correlation coefficients. Conversely, in WM-Ls (C, p < .01, overall Pearson coefficient = 0.99, 95% CI: 0.97, 1.00), thalamic (D, p < .01, overall Pearson coefficient = 0.83, 95% CI: 0.69, 0.98) and dGM ROIs (E, p < .01, overall Pearson coefficient = 0.82, 95% CI: 0.81, 0.84), positive associations were found between R2* values and myelin intensities. These associations were not significant in ROIs of shadow plaques. In the cortex, significant positive associations were found between R2* values and myelin intensity as measured by PLP (F, p < .01, overall Pearson coefficient = 0.57, 95% CI: 0.55, 0.58).

#### R2* values and iron content (Fig 4A–4E)

Iron intensity was significantly associated with R2* values in ROIs of NAWM (A, p<0.01, overall Pearson coefficient = 0.78, 95% CI: 0.76, 0.79), DWMI (B, p<0.01, overall Pearson coefficient = 0.42, 95% CI: 0.39, 0.45) WM-Ls (C, p<0.01, overall Pearson coefficient = 0.81, 95% CI: 0.66, 0.96) and dGM (D, p<0.01, overall Pearson coefficient = 0.86, 95% CI: 0.84, 0.88) and normal-appearing cortex (E, p<0.01, overall Pearson coefficient = 0.42, 95% CI: 0.40, 0.44). Conversely, in thalamic and shadow plaque ROIs this association was not significant.

#### Iron and myelin (LFB, PLP) content (Fig 5A–5E)

**Fig 5:**
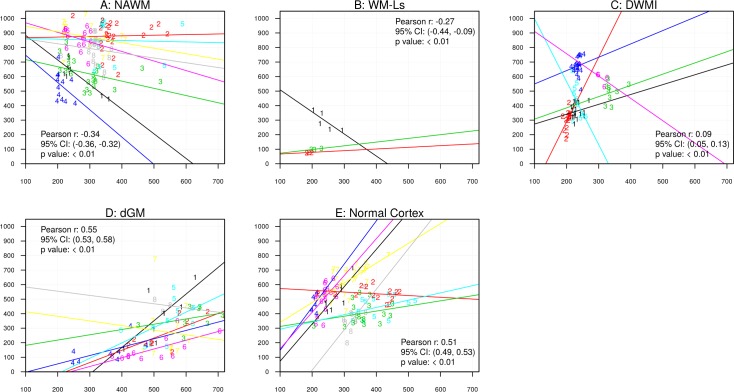
Scatter plots of myelin and iron intensity in ROIs placed in NAWM (A), WM-Ls (B), DWMI (C), dGM (D) and cortex (E). Data points are labeled with subject ID number, so that the same number indicates multiple measurements from the subject. Y-axes indicate myelin concentrations and x-axes indicate iron concentrations. In the fig: au = arbitrary units; dGM = deep gray matter, DWMI = diffuse white matter injury, NAWM = normal appearing white matter, WM-Ls = white matter lesions. Each different colored line represents data from a different sample.

Fig 5 shows associations between iron and myelin content. Myelin (LFB) intensity was significantly but inversely associated with iron intensity in ROIs of NAWM (A, p<0.01, overall Pearson coefficient = -0.34, 95% CI: −0.36, −0.32) and in WM-Ls (B, p<0.01, overall Pearson coefficient = -0.27, 95% CI: -0.44, -0.09). Conversely, associations were positive and significant in ROIs placed in DWMI (C, p<0.01, overall Pearson coefficient = 0.09, 95% CI: 0.05, 0.13) and dGM (D, p<0.01, overall Pearson coefficient = 0.55, 95% CI: 0.53, 0.58). In the normal cortex, a significant positive association between PLP and iron was found (E, p<0.01, overall Pearson coefficient = 0.51, 95% CI: 0.49, 0.53).

### Associations between R2* values and myelin/iron intensity over all different anatomical regions and across samples (Fig 6A–6C): secondary analysis

**Fig 6:**
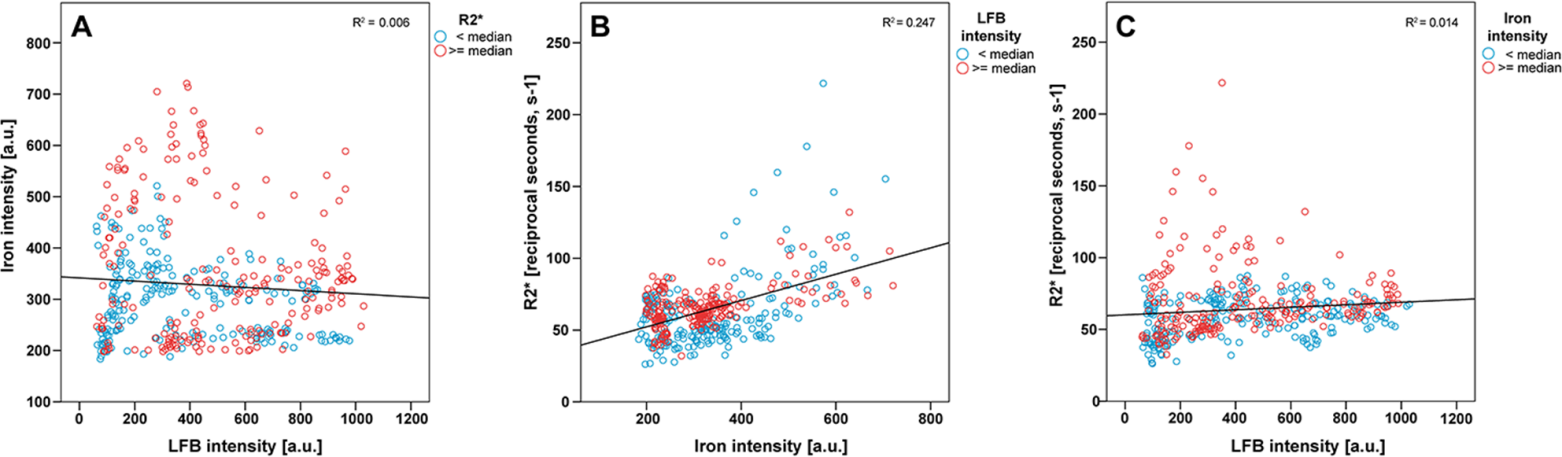
Scatter plots of LFB (myelin) and iron intensities (A), iron intensities and R2* values (B) and LFB intensity and R2* values (C) across the all anatomical regions and brains. Two variables are displayed in each of the scatter plots on the x- and y-axis, while the third variable has been dichotomized using its median and displayed using two different colors. Linear regression lines derive from all data points are depicted. Given R^2^ values correspond to the regression lines.

For this analysis only, myelin content of cortical ROIs was measured by LFB intensities in order to retrieve a continuum of myelin intensities across all anatomical regions. Fig 6A demonstrates the lack of association between iron and LFB intensities, when analyzed across all anatomical regions and all cases together. Fig 6B demonstrates the positive association between iron intensity and R2* values, which proved present in ROIs with both low (red circles) as well as high myelin densities (blue circles). Fig 6C depicts the relationship between LFB intensity and R2* values and suggests a weak overall positive association, more so in ROIs low in iron (blue circles).

In the linear mixed-effects model, no significant interaction between the two predictors, i.e., LFB and iron intensity, was observed (p = .06). Therefore, the interaction term was not included in the model. In this reduced model, no significant effect of the anatomical or pathological regions was observed (p = .81). R2* values increased by 0.13 (95% CI: 0.10–0.16, p < .001) with single unit increment of iron intensity, when LFB intensity was kept constant. R2* values increased by 0.02 (95% CI: 0.01, 0.03, p < .001) with single unit increment of LFB, when iron intensity was kept constant.

An overall summary of the findings in relation to pathophysiology is provided in Table 4.

**Table 4:** Summary of the findings.

**Regions**	Relation between myelin content and R2*	**Main biological factor**
NAWM	Weakly inversely associated	Myelin fiber directionality with respect to the B_0_.
DWMI	Weakly inversely associated	
WM-Ls	Positively associated	Residual healthy myelin content which is predominantly reflected by the quantity of iron in oligodendrocytes and fiber directionality.
Thalamus	Positively associated	
dGM	Positively associated	
Cortex	Positively associated	
Shadow Plaques	Not significantly associated	
**Regions**	Relation between iron content and R2*	**Pathophysiology**
NAWM	Positively associated	Likely driven by iron inside heathy oligodendrocytes.
DWMI	Positively associated	Iron content in healthy oligodendrocytes, scavenger cells, neurons, activated and senescent microglia.
WM-Ls	Positively associated	
Cortex	Positively associated	
dGM	Positively associated	
Thalamus	Not significantly associated	
Shadow Plaques	Not significantly associated	

## Discussion

Consistent with previous imaging studies [33, 43–45] in both healthy [33, 44] and diseased brains [43–45], we found that R2* values varied across brain regions of MS patients and were significantly associated with both myelin and iron intensities across the brain. dGM ROIs, followed by NAWM and DWMI ROIs, displayed the highest values of R2*. The distributions of R2* values found in our study are similar to those reported previously in control brains post mortem [10] and MS brains in vivo [39] and likely reflect both anatomical variations as well as disease-associated changes.

Complementary to previous reports [43–45] focused on brains of humans with^43,45^ and without MS [44], we show that in our MS samples; (1) the within samples associations between R2* and myelin/iron intensities vary between subjects and anatomical regions, and (2) the association between R2* values and iron intensity is stronger than the one between R2* values and myelin intensity, across and within individual anatomical regions.

We performed two types of statistical approaches. Given the small sample size, both analyses remain preliminary and descriptive. The primary analysis was based upon Pearson coefficients [42]. To properly weight the intra-case dependency of the measurements as done in these types of studies and to avoid confounding biases due to masked inter-case variability and due to the large degree of intra-case data correlation, we weighted individual cases’ Pearson coefficients by their standard error estimates. This methodology [42] allowed a more precise estimate and understanding of the consistency of results produced across cases. We also performed analyses across all cases using linear mixed-effects models and we present the output of this secondary analysis, recognizing the potential weaknesses associated to the small number of studied cases.

### Associations between R2* and myelin intensity

We quantified myelin using two methods, i.e., LFB and PLP staining and densitometry. LFB stains myelin phospholipids, while PLP is a major myelin protein and comprises the largest component of CNS myelin proteins (~50%)^39^. As seen previously [39], in our data DWMI showed a more conspicuous loss of LFB reactivity than PLP reactivity. This effect was also seen in shadow plaques, which showed a clear LFB reduction but were barely visible in PLP stains. Our overall experience is that PLP has a higher sensitivity for low myelin concentrations (like in the cortex). Instead, it becomes quickly saturated with higher myelin concentrations, thus not properly reflecting subtle differences in myelin density, like those one can find between NAWM and DWMI. On the other hand, LFB has a lower sensitivity but is well suited to reflect myelin density differences in highly myelinated areas. On the basis of these observations, we chose PLP densities for the neocortex alone, but have selected LFB for the comparison of R2* across all regions in our survey including the neocortex.

A high degree of heterogeneity in the relation between R2* and myelin content in non-lesional areas (either NAWM or areas of DWMI) was found within samples. These areas, although not necessarily showing an intact myelin content and normal thickness, may preserve the anatomical orientation of their fibers. The overall inverse association was weak, but significant. Care needs to be taken in interpreting these results since the overall Pearson coefficient was very low. This low value was determined by the averaging of the negative and positive associations found on each subject and it was reflected by the overall weak association seen with the linear mixed effects models. More than to the overall directionality of the association, one shall consider the inter-sample heterogeneity which likely end-resulted in lack of region-effect in the linear mixed effects models. To this end, our findings are in line with previous studies examining the heterogeneity of T2*/R2* values in the WM and their relation to myelin content in healthy brains [20]. ROIs in the superior corona radiata had significantly lower R2* than the cingulum. This difference was reflected by higher myelin content as well as more compact and coherently oriented fibers present in the cingulum [20]. Oh and collaborators also demonstrated that within the normally structured corpus callosum, the relative orientation between WM fibers and the B_0_ field predominately affects R2* contrast, suggesting that the major source of contrast in R2* is magnetic susceptibility [46]. This ex vivo evidence is supported by in vivo data demonstrating highly different R2* between fibers in close vicinity but with different orientations [47]. Both similarities in microstructural characteristics between fibers sharing the same orientation, as well as individual fibers orientation relative to the main magnetic field of the scanner explain changes in R2* in healthy WM [25]. The molecular bases of this orientation is ultimately ascribable to water content, the integrity of lipids vs proteins, iron contained in several protein among which—in vivo especially–dexohemoglobin [25]. Chemical exchange does not seem to play a role [25]. In our work, aside from the corpus callosum, ROIs were randomly distributed in the NAWM and DWMI. Since capturing areas with random myelin content and orientation, it is very likely that this random distribution has made the histology-MRI correlations heterogeneous. In interpreting our findings in MS brains one also needs to take into account that area of DWMI, although preserving the main directionality of their fibers, do exhibit a low degree of axonal damage and swelling [40]. Variable amounts of intra-axonal water accumulation are present in areas of DWMI and these, although not in large part, still contribute differently to variations in R2*. Notwithstanding the above considerations, it is also possible—and not mutually exclusive—that variability in the associations between myelin content and R2* in different regions of the brains does reflect difference of iron [25] content inside oligodendrocytes in these regions.

In line with our findings and hypothesized explanations in NAWM and DWMI, the associations between myelin content and R2* became direct and stronger when quantified in regions with expected lower myelin content and higher degree of anisotropy, i.e., dGM and cortex, WM-Ls and thalamic ROIs. Langkammer and collaborators [33] who did look at the relation between R2* and cortical myelin as measured by magnetization transfer ratio in healthy brains, did find a strong trend towards a significant association, thus resembling our findings. We speculate that in these regions, fiber shape and orientation are no longer the major source of magnetic susceptibility and myelin content per se plays the major role. In this notion, answering the fundamental question as to if intra-oligodendrocyte ferritin-bound iron rather than myelin quantity is the major determinant of this contrast immediately follows. Cumulatively, this degree of heterogeneity in association deepening upon different brain regions result into and overall weak association when looked at with the linear mixed effects models.

### Associations between R2* and iron intensity

Our findings on the associations between iron density and R2* in diseased brains are overall in agreement with those reported in healthy [33, 48] and diseased [49] brains. In NAWM/DWMI areas, iron/R2* and myelin/R2* relationships had opposite directions and the iron/myelin association was rather weak. In comparing the degree of association (i.e., weighted Pearson coefficient) between iron or myelin and R2*, one can see that iron has a stronger effect on R2* changes than myelin. We believe that these findings are due to the fact that in NAWM and DWMI where myelin still has some degree of preservation, the geometry of the fibers in addition to myelin content per se determines this association. Conversely, in dGM, WM-Ls and normal cortical regions, iron/R2* and myelin/R2* relationships were very similar in strength and directionality and the iron/myelin association was rather strong. These findings suggest a high degree of intra-correlation between the, albeit overall smaller than in DWMI, quantities of iron and myelin. We interpret our findings with intracellular (i.e., intact and remaining oligodendrocytes) ferritin-bound iron as being the major determinant of the susceptibility contrast in WM-Ls, cortex and dGM [50, 51]. It is certainly plausible that other sources of iron also affect R2* and that the absence or presence of this iron, combined to the intracellular one, explains part of the variance of the susceptibility contrast. The predominance of iron as source of R2* contrast was confirmed by the secondary analyses which found a significant association between R2* and iron density in regions with both low and high iron content.

### Associations between myelin and iron intensity

For a better understanding and interpretation of our data, we also investigated the associations between iron and myelin content. The results of this analysis suggested that different iron/myelin interplay exists in NAWM/WM-Ls areas compared to GM areas of MS brains. This difference resulted into an overall lack of association when the linear mixed effects models were used. In the NAWM where both myelin and iron were significant contributors of the R2* signal, the iron/myelin association was inverse but somehow weaker. The data agree with those reported by Langkammer and collaborators [33] who also reported a negative association between iron and myelin (as measured by magnetization transfer ratio values) in WM. The data is an indirect confirmation of the role of fiber orientation more than myelin quantity per se in affecting the susceptibility contrast. They are also in line with the concept that intra-oligodendrocyte iron, which increases as a function of time in NAWM, reaches a plateau around the age of 50 [52]. Similarly in WM-Ls, where iron and myelin both contributed to the R2* signal, the association between the two was inverse. We believe that given the paucity of the data, this association was mainly driven by data derived from one sample. Interestingly, data from the same sample showed no association between iron/myelin quantity and R2* changes. If one excludes this outlier, one can see that the iron/myelin association is rather direct in WM-Ls. In the core of chronic WM-Ls lesions, as well as in chronic lesions of the cortex, the overall amount of iron is very low. Oligodendrocytes, myelin and even microglia or macrophages tend to be no longer present and iron and ferritin are often detected within astrocytes and residual axons [31]. It is plausible that myelin debris is present in iron-load macrophages [30].

Conversely, in dGM, DWMI and normal cortical regions iron/myelin relationships were direct and rather stronger for the GM regions. Our work is an elegant demonstration that in the deep and cortical healthy GM myelin and iron contents does indeed run in parallel. Similarly, except for a few samples, there was an overall positive, although very weak, association between iron and myelin content in areas of DWMI. One may argue that in these areas, opposite to healthy WM at one end and to WM-Ls at the other end, some attempts of remyelination are still present. In that case, a higher influx of iron into oligodendrocytes may still be happening [53], and the overall quantity of iron is somehow a function of the preserved myelin.

For the purpose of this study, we did not track the cellular source of iron because outside the scope of the work and previously investigated in the work of our group [5, 30] and that of others [6, 7, 50–52].

### Study limitations

Several important study limitations need to be acknowledged. First, intrinsic to all studies of this type, is the effect of fixation on R2* values as well as on iron and myelin concentration. This effect is of particular relevance when one attempts to translate post mortem findings to in vivo applications. Formaldehyde fixation alters tissue properties by fostering protein cross-linking [54, 55] and reducing T2 values, compared to in vivo data [56, 57] ^7^. In MS brains, the effect of fixation has been reported to strength the association between T2-values ad myelin content. With respect to iron, instead, recent work has shown that there is indeed a substantial decrease of measurable iron content in brains after fixation [58]. This decrease does not necessarily alter the relation with R2* values (which remain unchanged), likely due to the fact that relative proportions of iron content remain unaltered [58].

Temperature is another important source of variability between in vivo and post mortem scanning [59], making our results not directly transferable to in vivo imaging. According to Curie’s Law, the paramagnetic part of the magnetic susceptibility is inversely proportional to temperature. This has recently been shown to lead to an approximately linear relationship between R2* and temperature[60]. While this linear scaling is likely to increase contrast values as the temperature drops from 37C to room temperature, thereby possibly counteracting the iron loss due to fixation, it is unlikely to create new features in the image. Therefore, we believe that the R2* changes observed in our room temperature, fixed-brain imaging will correspond to similar changes, but possibly of lesser contrast, in in vivo imaging.

### Practical applications and conclusions

Overall our study provides preliminary but solid evidence of the complex source of susceptibility contrast in MS brains. Although our sample size is small, i.e., only eight cases, our data are a respectable demonstration that variations in R2* are affected differently by myelin structure and iron content in different regions of MS brains. Our overall findings suggest that the association between iron/myelin and R2* is not univocal. We provide additional and different demonstration that R2* values are affected by both myelin and iron contents. Changes in the content of one may affect the content of the other. In translating our results in vivo, care needs to be taken when interpreting MRI data in MS patients and careful consideration of the anatomical and pathological region needs to be done in order to properly assess the meaning of T2*/R2* changes.
